# Missing Diagnoses during the COVID-19 Pandemic: A Year in Review

**DOI:** 10.3390/ijerph18105335

**Published:** 2021-05-17

**Authors:** Héctor Pifarré i Arolas, Josep Vidal-Alaball, Joan Gil, Francesc López, Catia Nicodemo, Marc Saez

**Affiliations:** 1 Centre for Research in Health Economics, Universitat Pompeu Fabra, 08002 Barcelona, Spain; hector.pifarre@upf.edu (H.P.iA.); francesc.lopez@cmail.cat (F.L.); 2 Health Promotion in Rural Areas Research Group, Gerència Territorial de la Catalunya Central, Institut Català de la Salut, 08272 Barcelona, Spain; 3 Unitat de Suport a la Recerca de la Catalunya Central, Fundació Institut Universitari per a la Recerca a l’Atenció Primària de Salut Jordi Gol i Gurina, 08007 Barcelona, Spain; 4 Faculty of Medicine, University of Vic-Central University of Catalonia, 08500 Vic, Spain; 5 Barcelona Economic Analysis Team, Department of Economics, Universitat de Barcelona, 08007 Barcelona, Spain; joangil@ub.edu; 6 Centre of Organisation, Department of Primary Economics, University of Oxford, Oxford OX1 3UQ, UK; catia.nicodemo@economics.ox.ac.uk; 7 Research Group on Statistics, Econometrics and Health, Department of Economics, Universitat de Girona, 17004 Girona, Spain; marc.saez@udg.edu; 8 Epidemiology and Public Health Networking Biomedical Research Centre, 28029 Madrid, Spain

**Keywords:** indirect impact, delayed diagnoses, COVID-19, SARS-CoV-2, access to healthcare

## Abstract

The COVID-19 pandemic has had major impacts on population health not only through COVID-positive cases, but also via the disruption of healthcare services, which in turn has impacted the diagnosis and treatment of all other diseases during this time. We study changes in all new registered diagnoses in ICD-10 groups during 2020 with respect to a 2019 baseline. We compare new diagnoses in 2019 and 2020 based on administrative records of the public primary health system in Central Catalonia, Spain, which cover over 400,000 patients and 3 million patient visits. We study the ratio of new diagnoses between 2019 and 2020 and find an average decline of 31.1% in new diagnoses, with substantial drops in April (61.1%), May (55.6%), and November (52%). Neoplasms experience the largest decline (49.7%), with heterogeneity in the magnitudes of the declines across different types of cancer diagnoses. While we find evidence of temporal variation in new diagnoses, reductions in diagnoses early in the year are not recouped by the year end. The observed decline in new diagnoses across all diagnosis groups suggest a large number of untreated and undetected cases across conditions. Our findings provide a year-end summary of the impact of the pandemic on healthcare activities and can help guide health authorities to design evidence-based plans to target under-diagnosed conditions in 2021.

## 1. Introduction

The COVID-19 pandemic has directly affected the health of most people infected with the SARS-CoV-2 virus but has also indirectly affected the rest of the population due, in part, to the ongoing and substantial disruption and strain on the normal functioning of healthcare systems. The spread of the disease has rapidly arrested and changed daily healthcare practice through, among other channels, significant reductions in hospital admissions [1,2,3], non-COVID-19 non-critical elective procedures [4], and emergency department visits [5]. These reductions in the provision of healthcare services reflect healthcare systems reaching capacity constraints as well as patients deferring or avoiding care out of fear of contracting the disease [6], with severe health consequences [7,8,9].

We study the indirect effect of the pandemic on new detected disease instances through the variation in diagnosed cases in the Catalan health administrative region of Central Catalonia, Spain. The Spanish experience is of particular interest, given that it has been one of the countries most severely impacted by the pandemic worldwide [10], in the context of a developed country with a highly accessible and largely free public healthcare system. Within Spain, Catalonia has been one of its most affected regions, and the particular area we study has also been amongst the worst hit within Catalonia. As of 4 December, Spain has experienced 3559 COVID-19 cases per 100,000, Catalonia 4053, and Central Catalonia 4294 (see Section 2).

Our work complements and extends existing evidence on the declines in new diagnoses during the first wave of the pandemic in the early months of 2020 [11]. We provide a complete overview of the changes in new diagnoses throughout the pandemic year, and document the monthly declines, which mirror the intensity of the pandemic, and the accumulated effect on new diagnoses across all ICD-10 codes [12]. The length of the period studied offers unique leverage in capturing the potential recovery effect on diagnoses in the months with lower COVID-19 incidence, which can inform upcoming such wanes in pandemic waves.

## 2. Materials and Methods

### 2.1. Population

The population studied consists of 417,706 patients, 62,722 (15%) children under 14 and 354,984 adults (85%), of which 44,034 are over 65 (for the year 2019). Our data captures the administrative records of all patient visits with the public Catalan primary healthcare teams (PCTs) in Central Catalonia affiliated with the Catalan Institute of Health, representing 86% of all PCTs in the region, from 1 January 2019 through 20 November 2020. We study all channels of patient visitation, including both face-to-face (at the primary healthcare center or at the patients’ residence) and telemedicine interactions, comprising of telephone consultations and eConsultations, an asynchronous internet-based telemedicine service between patients and primary care healthcare professionals. We compare a baseline of January through 20 November in 2019 to the same period in 2020. In 2019, through 20 November, there were 1,558,314 interactions by 296,703 unique individuals, 15.4% of which were via telemedicine. By the same time in 2020, interactions have risen to 1,604,891 from 299,907 unique individuals, while the share of telemedicine increased to 56.2%.

### 2.2. The Catalan Healthcare System

PCT records are well-suited to study changes in new diagnoses given the preeminence of the public health system in Catalonia and the central role of PCTs in the provision of healthcare services. The Catalan national healthcare system is publicly financed and provides universal health coverage free at the point of delivery, with modest co-payments for medications, to residents of Catalonia [13]. While around one fourth of Catalans attain additional private health insurance [14], they retain their right to access public healthcare. A defining characteristic of primary care services in Spain is that they are the first point of contact with healthcare services for the population [15] and host recorded diagnoses using an integrated electronic health record system that includes hospital diagnoses [16].

### 2.3. New Diagnoses

Based on visit level patient data from 2019 and 2020, we identify new diagnoses via two approaches. We flag a diagnosis as new if within the list of diagnoses associated to a given patient’s visit with the PCT, there is a diagnosis that is not in the list of preexisting active diagnoses of the patient. We also classify diagnoses as new if they are added to the list of active diagnoses across patient visits, between two consecutive visits. In this way, we can detect new diagnoses for patients with multiple or single visits with PCT throughout the year.

### 2.4. COVID-19 Cases

To describe the association between the intensity of the pandemic and the decline of diagnoses over time, we report the number of COVID-19 cases in the region during the period under study. Official reports on COVID-19 cases throughout the pandemic reflect incidence and, to an extent, the availability of tests to confirm the infection. As a result, during the so-called second wave of the pandemic (the resurgence of cases after widespread decline in the summer of 2020), official counts report far more cases than those accounted for during the early months of the pandemic. For this reason, we have opted to provide incidence measures based on our primary care data. We identify incidence as the counts of new diagnoses with COVID-19 codes (ICD-10 code B34.2 during the first months of the pandemic, followed by ICD-10 code U07.1). We record new diagnoses following the same procedure explained above and used for other conditions.

When we compare Central Catalonia to the rest of Catalonia and Spain in terms of accumulated COVID-19 incidence, we compute incidence rates from official confirmed COVID-19 cases [17,18] and the latest population counts available at the Spanish [19] and Catalan Institute of Statistics [20], for years 2020 and 2019, respectively.

### 2.5. Statistical Analysis

We follow the approach in related work [1] to test the significance in diagnoses reductions for both the decline in overall diagnoses and the chapter-specific reductions. We similarly assume that monthly diagnoses counts are drawn from two separate diagnoses distributions, for 2019 and 2020. We perform a paired (Welch) t-test on the differences of the means between the two diagnoses distributions, and report 95% confidence intervals. For ease of interpretation, results are presented in ratios over 2019 counts. To account for multiple hypothesis testing of the categories of diagnoses, we also report (denoted as * on the *p*-value, * *p* < 0.05) whether the results remain significant applying a Bonferroni correction to significance levels. Given that we conduct 29 distinct hypothesis tests (21 chapters, 6 subchapters, 2 periods), we test each hypothesis at a significant level of α = 0.00172. All statistical work was performed with R, version 4.0.5.

### 2.6. Diagnoses Counterfactuals

In Central Catalonia alone there were, from March to 20 November in 2020, 1748 deaths in the 60+ age group [18]. These are individuals who might have otherwise contributed new diagnoses, and thus been partially responsible for a decline in new diagnoses. To assess their potential role in our findings, we provide an estimate of the possible number of lost new diagnoses to this group. We calculate, for each of the age groups (“60–69”, “70–79”, “80–89”, “90+”), the age-specific distribution of new diagnoses in 2020 during the period under study. Then, for each death, we assume they would have had as many new diagnoses as the last (highest) quartile of their age-specific new diagnoses distribution.

## 3. Results

### 3.1. Main Results

From January through 20 November 2019, there were a total of 867,716 new diagnoses across all ICD-10 groups recorded in Central Catalonia’s PCTs. In direct contrast, excluding COVID-19 cases, during the same period for 2020, there has been 598,120 new diagnoses, resulting in an overall ratio of 0.689 new diagnoses in 2020 per 2019 diagnoses, or a 31.1% reduction (* *p* < 0.05, 95% CI 0.52–0.85); within major pandemic months (March through November 2020), we find a 37.1% decline (* *p* < 0.05, 95% CI 0.47–0.78). The results reported on the changes in diagnoses in this section are tested against a null of no change.

**Figure 1:**
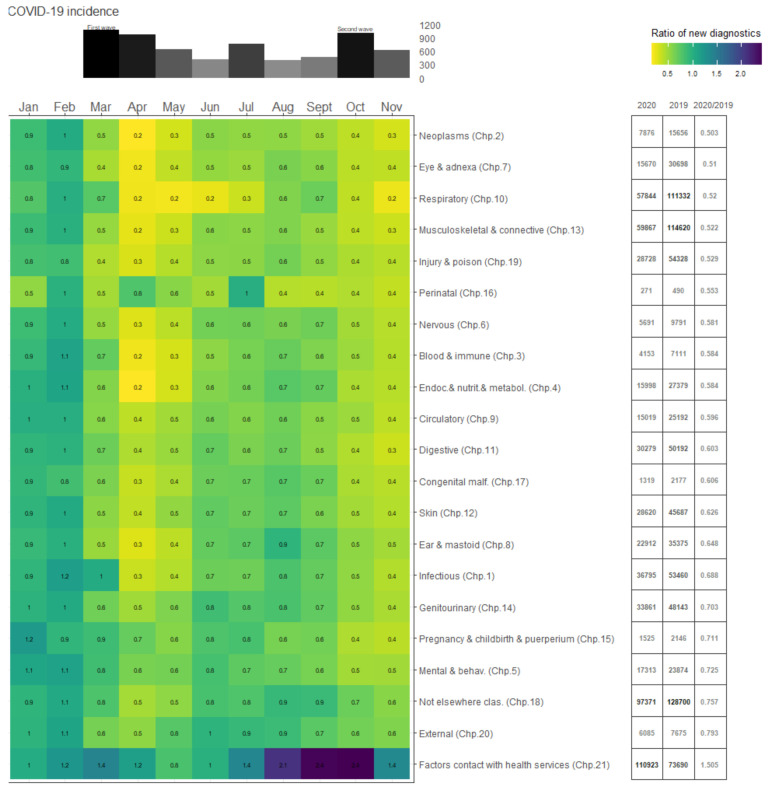
The heatmap presents, by month (x-axis), the ratio of new diagnoses in 2020 compared to new diagnoses in 2019, by ICD-10 groups (y-axis). Numerical values for the monthly ratios are provided for each month and ICD-10 group. Severe drops in diagnoses in 2020 are more yellow, similar diagnoses are in deep green, while increases in diagnoses are in purple. Monthly COVID-19 incidence per 100,000 for the region is plotted above the heatmap. The three columns to the right of the heatmap present counts of diagnoses by ICD-10 group in 2020, 2019, and the ratio of 2020/2019, respectively.

Figure 1 presents monthly ratios of new diagnoses in 2020 compared to new diagnoses in 2019 by ICD-10 chapter, with months as columns and chapters as rows. Severe drops in diagnoses in 2020 appear yellow, similar diagnoses are in green, while increases in diagnoses present as purple. Monthly COVID-19 incidence per 100,000 are presented above the heatmap. Columns to the right of the heatmap report counts of diagnoses by ICD-10 chapter in 2020, 2019, and the ratio of 2020/2019, respectively. Across almost all ICD-10 groups, the monthly trends in the ratios of new diagnoses closely mimic COVID-19 incidence (Figure 1, bar plot above the heatmap), with steeper declines during the peaks of the first wave of the pandemic (March through June 2020) and, to a lesser extent, the second wave (October through November 2020). The largest declines in new diagnoses in 2020 occurred in April (61.1% reduction) and May (55.6% reduction), followed by November (52% reduction). Even in months where reduction in diagnoses drop slightly (see, for instance, perinatal diagnoses in July in Figure 1), new diagnoses do not recover the year’s lost diagnoses—that is, there is no “catch up” by the end of the year.

Neoplasms (ICD-10 Chapter 2) experience the largest drop in new diagnoses, with nearly half the number of new diagnoses (49.7%; * *p* < 0.05, 95% CI 0.33–0.68) with respect to the 2019 baseline. We find the smallest declines for external causes (ICD-10 Chapter 20), with a 20.7% reduction (not significant at *p* < 0.05, with *p* = 0.14, 95% CI 0.51–1.08). The only group for which there are increased new diagnoses are for factors influencing health status and contact with health services (ICD-10 Chapter 21), with 50.5% additional new diagnoses (not significant at *p* < 0.05, with *p* = 0.052, 95% CI 0.99–2.02). This is largely explained by the fact that the category captures new diagnoses associated with individuals in contact with COVID-19 cases.

### 3.2. Cancer Diagnoses

Among all conditions, an early diagnosis is of particular importance for cancer. An examination across cancer types reveals that, among the four types of cancers with the highest incidence in 2019, the decline was smaller for breast (17.9%, not significant at 0.05, with *p* = 0.109, 95% CI 0.54–1.05) and bowel cancer (31.7%; * *p* < 0.05, 95% CI 0.49–0.87) compared to benign neoplasms (55.8%; * *p* < 0.05, 95% CI 0.26–0.62) and sarcomas and mesothelial cancers (50.2%; * *p* < 0.05, 95% CI 0.24–0.70). Breast and bowel cancers are high incidence types subject to extensive screening programs, whereas sarcomas and mesothelial cancers are not.

### 3.3. Socieconomic Gradient

New diagnoses from ICD-10 codes associated with the provision of health-oriented social services (ICD-10 Chapter 20, sub-chapters 10 and 15) have close to halved with respect to 2019 (47.2% and 45% reductions respectively, * *p* < 0.05, 95% CI 0.35–0.70 and * *p* < 0.05, 95% CI 0.37–0.73). Given that lower income groups disproportionately use these services, this might indicate that disadvantaged groups could have experienced relatively larger declines in their access to healthcare services.

## 4. Limitations

### 4.1. Missing Data

There are visits to PCT with no reported associated diagnoses (20.51%). This may affect our results insofar as the number of visits with missing data is relatively more numerous in 2020. In 2019, 18.46% of the visits had no reported associated diagnoses, whereas in 2020, there were 22.50% visits with missing data. Thus, even if missing data instances were relatively more frequent in visits associated with new diagnoses, missing data may only have a limited impact on the reported results.

### 4.2. COVID-19 Under-Counting

Our approach to measuring COVID-19 incidence is likely to suffer from under-counting, given the limitations on the availability of tests, especially during the early months of the pandemic. However, our strategy is consistent with the method we use to identify other new diagnoses; thus, we believe that it provides a sensible way to identify the trends in COVID-19 incidence throughout the period under study.

### 4.3. Deaths and Missing Diagnoses

We find that at most, 10,609 diagnoses may have been lost to COVID-19 deaths. This represents a comparatively small fraction (4%) of the 269,596 decline in diagnoses in 2020 with respect to 2019, and thus may only have a limited influence on our results. This exercise is conservative given that it not only assumes deaths are exclusively among those with higher potential diagnoses, but also that deaths occur at the beginning of the year.

## 5. Discussion

Notwithstanding the possible changes in incidence, our findings on the declines in new diagnoses across all diagnosis groups suggest a large number of untreated and undetected cases across conditions. Our findings indicate that, while new diagnostic counts partially recovered during the months with relatively lower COVID-19 incidence, there remains a large reduction in the total number of new diagnoses. The importance of untreated and undiagnosed conditions and the adequate prioritization of care has been highlighted throughout the pandemic [21,22,23]—a problem that is likely to manifest in greater demand of healthcare services in the coming months [24,25]. The overall decline in new diagnoses reflects both changes in the incidence of the different diagnosis groups and the capacity of the healthcare system to identify and address the healthcare needs of the population. For some diagnosis groups, such as neo-plasms, the reduction in new diagnoses is likely mostly due to a decline in testing procedures. Indeed, existing screening programs for cancer types with high incidence, such as breast and/or bowel cancers, were interrupted during the peak months’ first wave of the pandemic and resumed mid-June 2020. However, our results indicate that bowel and breast cancers experienced lower declines during the pandemic (breast, 17.9%, not significant at *p* < 0.05, with *p* = 0.109, 95% CI 0.54–1.05, and bowel 31.7%; * *p* < 0.05, 95% CI 0.49–0·87), suggesting that screening programs successfully recuperated part but not all of the new diagnoses not detected during the worst months of the pandemic. This is in line with findings from the Netherlands [26]. Other cancer types, outside the scope of large screening programs (sarcomas and mesothelial cancers), may remain more substantially under-diagnosed (50.2%; * *p* < 0.05, 95% CI 0.24–0.70). In the case of other diagnosis groups, such as mental disorders, separating between incidence and detection is more difficult. Given the reports of increased incidence of mental and behavioral conditions during the pandemic [27,28], the moderate reduction in new diagnoses may mask a higher proportion of undetected and untreated cases.

The steep reductions in the number of cancer diagnoses across countries have attracted considerable attention and concern [7,8,23,26,29], while more research is needed to establish the subsequential health consequences. Although the brunt of the health impacts of delays in cancer diagnoses has yet to manifest, there exist early evaluation exercises that attempt to predict it [7,8]. These contributions combine simulated scenarios for the recovery of missing cancer diagnoses and the estimated mortality impact of diagnoses delays on different types of cancers. These findings suggest a predicted 5–9.6% increase in the number of deaths, depending on the type of cancer, and up to 63,229 years of life lost to cancer in the UK alone. In addition, there is evidence that patients already diagnosed with some cancers are more likely to cancel oncology appointments for fear of COVID-19 contagion [29], which may further increase the impacts of the pandemic. It is thus crucial to identify, in each region, which cancers have the highest potential backlog in diagnoses and treatment to design evidence-led policy interventions. The delay in diagnoses of type 1 diabetes throughout the pandemic months has also raised significant concerns [30,31,32]. Timely diagnosis of type 1 diabetes is important to avoid diabetic ketoacidosis, an acute complication often associated with delayed diagnosis of the disease [33]. Findings from the early months of the pandemic in a 2020 report underline not only a reduction in diabetes diagnoses but also relatively worse clinical conditions in the newly detected cases [30,31,32]. There is, however, mixed evidence on whether, at least for the first months of the pandemic, the worsened health status might be due to the delay in referrals and diagnoses or due to complications associated with the COVID-19 infection [34], which could be in theory subsequently recovered in later months. Our results indicate that, across conditions, there was no such recovery of diagnoses by year’s end (31.1% reduction overall, * *p* < 0.05, 95% CI 0.52–0.85), suggesting that the negative impact of delayed diagnosis is likely to accumulate, independent of the other direct and negative impacts of COVID-19.

In this pandemic-constrained context, more healthcare systems across regions and countries have resorted to telemedicine, including phone- and internet-based consultations, to face the increased demand for healthcare services while complying with containment policies [35] and public health concerns of the population. This has also been the case in the context of our population, where we find that the share of telemedicine-based visits has increased to 56.2%, from 15.4% in the pre-pandemic year. A recent evaluation of the profiles of telemedicine users in Catalonia, Spain [36], indicates that the growth of telemedicine is also linked to increased utilization by new societal groups. Prior to the pandemic, the average profile of a telemedicine user in Catalonia was a middle-aged high-income female from an urban area. During the pandemic, underrepresented groups, including lower income individuals and patients residing in rural areas, are increasingly accessing healthcare through telemedicine. Patient clinical complexity appears to be lower during the pandemic months too, as the average telemedicine user during the pandemic more closely resembles the average user of the Catalan public healthcare system rather than the more skewed pre-pandemic average type of user. Telemedicine appears to be servicing a broader and more population-representative class of healthcare concerns, raising concerns over the adequacy of the healthcare provision.

Given the changing characteristics of the users, and the conditions treated, an extensive literature has studied the potential implications for the quality of provision and has found mostly positive results [35]. A particular area where telemedicine might face limitations, however, is in generating certain disease diagnoses that can require more involved assessments, such as cancer. However, it has also been pointed out that telemedicine may, in fact, be part of the solution to the cancer backlog [37]. Doctors can visit more patients in a day, and decreased diagnosis capacity can be alleviated via the use of artificial intelligence-powered diagnosis tools. Taken together, most of the arising evidence points to increased adoption of telemedicine as a tool that has helped navigate the challenging pandemic months and is certain to become a key service in pandemic preparedness [38].

It is well-established that the pandemic has had a relatively graver direct effect on disadvantaged populations [39,40,41]. In addition, access to care may have also been more severely limited for lower income groups and minorities. The disruption in healthcare services has been documented as more severe in poorer areas [1]. Furthermore, the cancellation of appointments and surgical procedures may have disproportionately affected individuals belonging to minority and other identity groups that already faced inequalities in healthcare access previously [42]. Our results are well-aligned with these findings, although they are limited to specific health-oriented social services. A direct assessment of socioeconomic inequalities in access to healthcare in the region is of clear importance. However, such an exercise is beyond the scope of our study. Taken together, the existing evidence for the relatively larger health impacts on disadvantaged socioeconomic groups suggests urgent creation of programs that focus on specific vulnerable sub-populations, as these groups may have experienced a double burden of negative health effects, stemming from the direct and indirect effects of the pandemic.

## 6. Conclusions

Our article provides a complete overview of the decline in new diagnoses throughout the pandemic (as of 20 November) that we hope will help guide health authorities to design evidence-based policies to target under-diagnosed conditions in 2021. Until normal operations are fully restored, under-diagnosed cases will continue to accumulate. Our findings may help identify diagnosis groups that have been particularly impacted by the disruptions caused by the pandemic, although additional efforts are needed to complement existing evidence to identify the sub-populations that have been more severely affected. So far, the current body of evidence points towards vulnerable socioeconomic populations.

While we believe that our findings might help guide further scrutiny in other countries and regions, we also encourage context-based interpretation of the results presented here. When considering how well the findings export to other countries, a key consideration to note is that the Catalan healthcare system is public and free access, two factors which lower barriers to healthcare access and which may vary across contexts [43].

## Data Availability

The data was extracted from the computerized medical records of the Information System for Primary Care Services (SISAP in Catalan) of the Catalan Institute of Health, in Barcelona, Spain. These data are not publicly available, and restrictions apply to the availability of the data used for the current study. These data are available upon reasonable request addressed to the Catalan Institute of Health. A de-identified and appropriately redacted random sample of the data and code are available at our github repository: https://github.com/HPiArolas, accessed on 10 December 2020.
